# Prevalence of Generalized Anxiety Disorder Among Five European Countries Before and During COVID

**DOI:** 10.1192/j.eurpsy.2024.669

**Published:** 2024-08-27

**Authors:** D. Karlin, S. J. Suponcic, N. Chen, C. Steinhart, P. Duong

**Affiliations:** ^1^MindMed, New York; ^2^Value & Access Advisors, LLC, Tallahassee; ^3^Cerner Enviza, an Oracle company, Kansas City, United States

## Abstract

**Introduction:**

Globally, there is a mental health crisis, and anxiety is the most prevalent mental health condition. However, the impact of the COVID-19 pandemic (COVID) on generalized anxiety disorder (GAD) prevalence has not been quantified across European countries, and such impact could establish a new baseline of GAD estimates in European countries.

**Objectives:**

To assess GAD by severity level before and during COVID in 5 European countries, using the 7-Item GAD Questionnaire (GAD-7).

**Methods:**

Adults (age 18+) in France, Germany, UK, Italy, and Spain completed a short survey in May 2020 to assess the impact of COVID on their mental health. All respondents had previously participated in the National Health and Wellness Survey, a nationally representative survey of the adult general population in each country, before COVID (December 2019–March 2020). In both surveys, respondents completed the GAD-7. GAD symptoms were defined by GAD-7 score as mild (5-9), moderate (10-14), and severe GAD (≥15). Positive screen was defined as GAD-7 score ≥10. Positive screen and GAD symptom severity prevalence were reported for the pooled European sample and by country, both before and during COVID. Chi-square and McNemar’s tests were used to evaluate the difference in GAD severity across countries and changes over baseline in GAD positive screen during COVID. P-values were reported for both tests.

**Results:**

In total, 2401 adults were included in analysis (France, n=482; Germany, n=487; UK, n=487; Italy, n=474; Spain, n=471). Prior to COVID, 311 (13%) screened positive for GAD, with 208 (9%) moderate and 103 (4%) severe in the pooled European sample. During COVID, the distribution of GAD symptoms almost doubled, as 576 (24%) screened positive for GAD, and shifted towards greater severity with 337 (14%) moderate and 239 (10%) severe in the pooled European sample (** Figure 1**). Before COVID, the prevalence of positive screen ranged from 11% (France, Germany, Spain) to 16% (UK). Statistically significant increases in positive screen over baseline levels were observed across all countries (p<0.01), except Germany. Spain was the most impacted by COVID (increase: 16%), followed by Italy, France, and UK (increase: 14%, 12%, and 9%, respectively). Germany was the least affected, overall (increase: 4%) (** Figure 2**).

**Image:**

**Figure:**
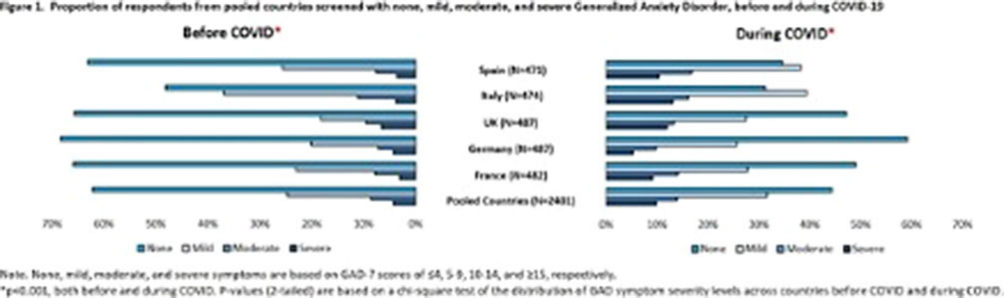

**Image 2:**

**Figure:**
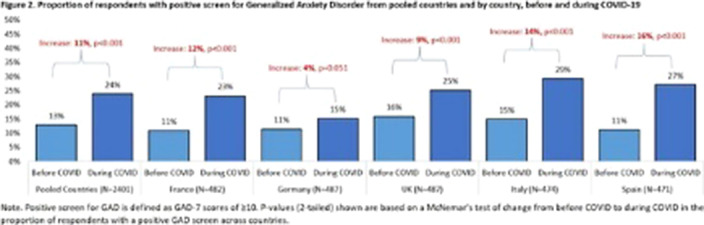

**Conclusions:**

During COVID, estimates of positive screen for GAD increased substantially to 24% across 5 European countries. Surges in positive screen and GAD symptom severity were observed in all 5 countries, with more profound impact in Spain, Italy, France, and UK. With new baseline GAD estimates, the country-specific data of COVID impact on GAD could help to inform appropriate allocation of mental health resources.

**Disclosure of Interest:**

D. Karlin Employee of: MindMed, S. Suponcic Shareolder of: Eli Lilly, Stryker, Abbott, Amgen, Consultant of: MindMed, Becton Dickinson Company, CSL Behring, N. Chen Consultant of: MindMed, C. Steinhart Employee of: MindMed, P. Duong Employee of: MindMed

